# Neurocytoma arising from a mature ovary teratoma: a case report

**DOI:** 10.1186/s13000-015-0406-x

**Published:** 2015-09-17

**Authors:** Juan-Han Yu, Lian-He Yang, Xu-Yong Lin, Shun-Dong Dai, Xue-Shan Qiu, En-Hua Wang

**Affiliations:** Department of Pathology, the First Affiliated Hospital and College of Basic Medical Sciences, China Medical University, Shenyang, China

## Abstract

Central neurocytoma/extraventricular neurocytoma is a central nervous system (CNS) tumor composed of uniform round cells with neuronal differentiation. The typical lesions of central neurocytoma/extraventricular neurocytoma are at the interventricular foramen of the lateral ventricles (central neurocytoma) or brain parenchyma (extraventricular neurocytoma). Mature teratoma is a benign germ cell tumor commonly found in young women. Herein, we report a 24-year-old female with neurocytoma in a mature teratoma of the right ovary. The histological examinations showed mature epidermis, skin appendages, adipose and bone tissues in the tumor; microscopic foci of immature cartilage tissues were also found in some parts. In addition, massive solid sheets and uniform round tumor cells were found in the neuroectodermal tissues, with the formation of neuropil-like islands. Immunohistochemical examinations showed that the tumor cells were synaptophysin- and NeuN-positive but GFAP-negative. Based on these findings, the woman was diagnosed with neurocytoma arising from mature ovary teratoma, with microscopic foci of immature cartilage tissues. This is the fourth case report of neurocytoma outside the CNS to date.

## Background

Central neurocytoma/extraventricular neurocytoma is a low-grade tumor with neuronal differentiation that occurs in the central nervous system (CNS), and histologically corresponds to WHO grade II. This tumor is predominantly found in young adults, and the prognosis is generally good. Central neurocytoma usually occurs at the lateral ventricles, while extraventricular neurocytoma could occur at any extraventricular regions in the CNS. The histological features and immunohistochemical phenotypes of central and extraventricular neurocytomas are similar: both are composed of round cells with homogeneous morphology and neuronal differentiation, and the tumor cells are positive for neuronal markers, such as synaptophysin, and sometimes NeuN [1, 2].

Mature teratoma is a benign germ cell tumor of the ovary, commonly found in reproductive women, which is composed of mature tissues from two or three germ layers. Somatic-type tumors arising from dermoid cysts are very rare, while tumors of CNS arising from mature teratoma are even rarer. Previous studies have reported that tumors of CNS arising from mature teratoma are generally from glial cells or primitive neuroectodermal cells [3]. Herein, we report a 24-year-old female with neurocytoma arising from a mature teratoma of the right ovary.

## Case presentation

**Fig. 1:**
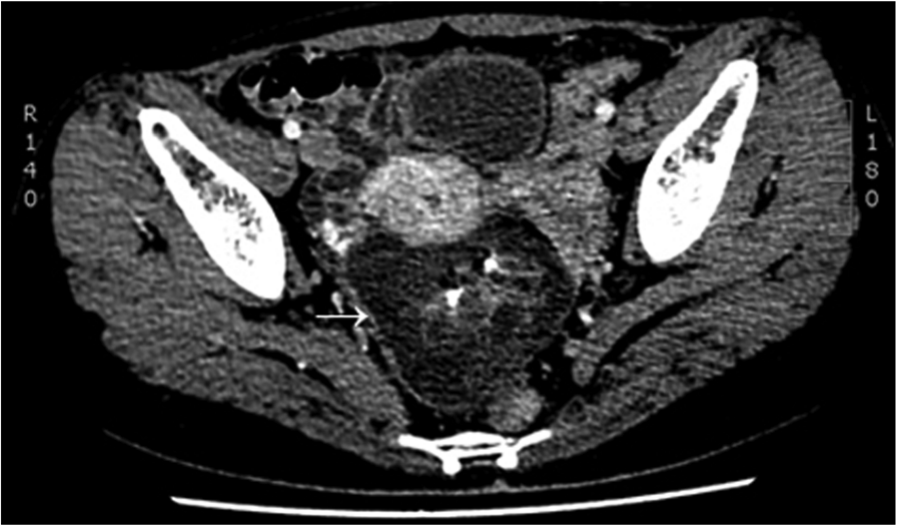
A mass with mixed-density is seen posterior to the uterus. The anteroposterior and transversal diameters of the mass were about 7.3 and 9.0 cm

A 24-year-old female was admitted to our hospital with amenorrhea for 10 months, and a pelvic mass for 15 days. She had regular menstrual cycles; however, the menstruation stopped 10 months earlier for no obvious reasons. Physical examination found no abnormalities. CT examination showed a mass posterior to the uterus with mixed density. The anteroposterior and transversal diameters of the mass were about 7.3 and 9.0 cm, respectively (Fig. 1). The woman was initially diagnosed with teratoma, and was surgically treated.

## Materials and methods

The resected specimens were fixed with 10 % neutral-buffered formalin and embedded in paraffin blocks. Tissue blocks were cut into 4-μm slides, deparaffinized in xylene, rehydrated with graded alcohols, and immunostained with the following antibodies: cytokeratin (CK, AE1/AE3), glial fibrillary acidic protein (GFAP, GA-5), synaptophysin (SP11), NeuN (A60) and Ki67 (MIB-1) (MaiXin, China). Sections were then stained with a streptavidin-peroxidase system (KIT-9720, Ultrasensitive TM S-P, MaiXin, China). The chromogen used was diaminobenzidine tetrahydrochloride substrate (DAB kit, MaiXin, China). All samples were counterstained with hematoxylin, dehydrated, and mounted. For the negative controls, each sample was incubated with PBS, instead of the primary antibodies.

## Results

**Fig. 2:**
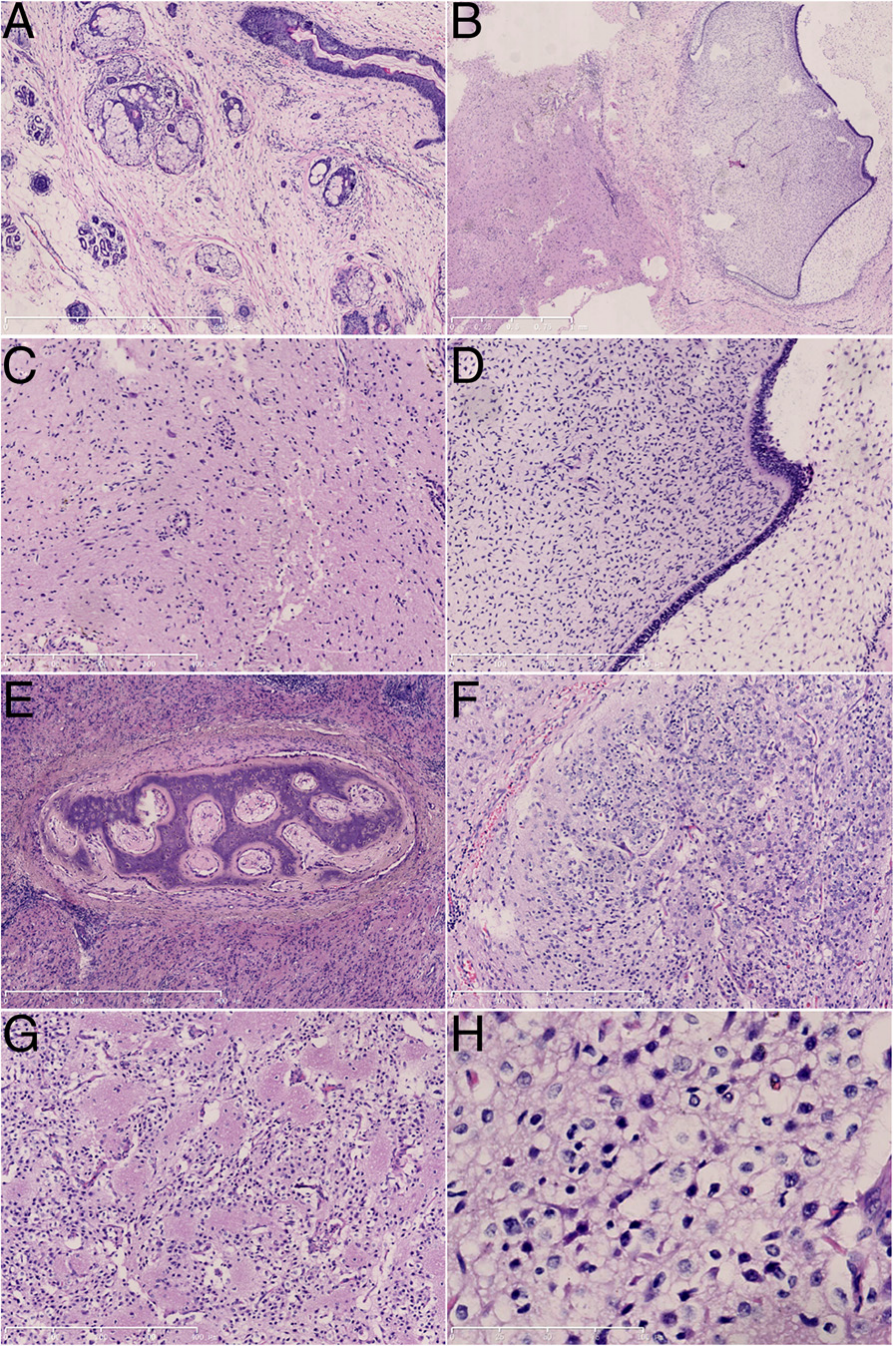
**a** Mature epidermis, cutaneous appendages, and adipose tissues in the tumor tissues; **b** neuroectodermal tissues and mesenchyme at local regions of the tumor; **c** (magnified figure of **b**): mature CNS tissues including neurons, glial cells, and ependymal tubules; **d** (magnified figure of **b​**) the mesenchyme tissues are immature cartilages (the immature cartilages are only found here); **e** mature bone tissues among the neurocytoma tissues; **f** clear boundaries with the surrounding neuroectodermal tissues; **g** neuropil-like islands formed by the tumor cells; and H: densely arranged uniform round cells with perinuclear halos with oligodendroglioma-like honeycomb appearance. **a** and **b** are at low magnification, **c**-**g** are at moderate magnification, and **h** is at high magnification

**Fig. 3:**
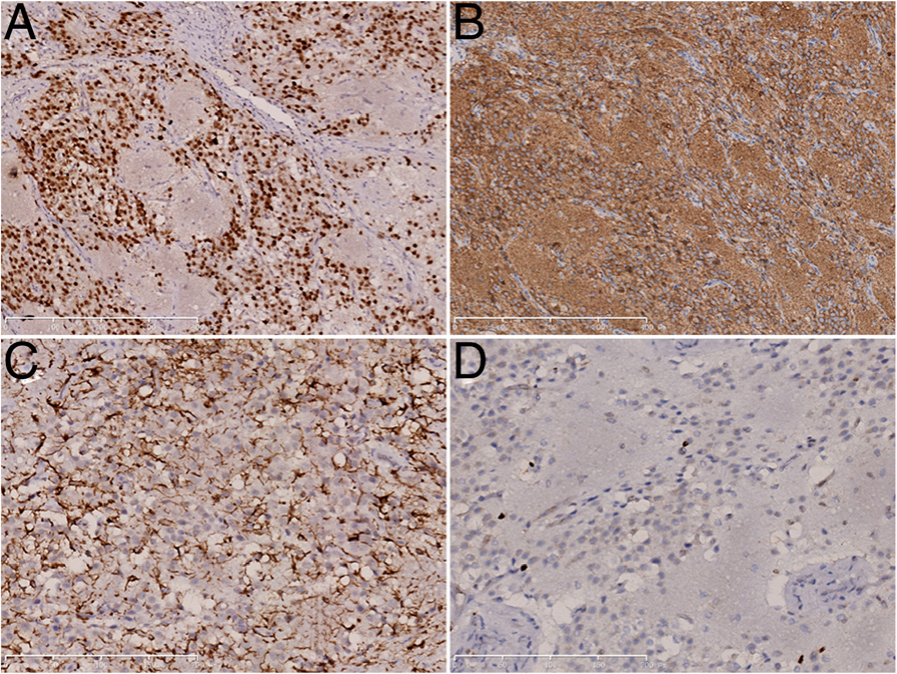
**a**: Immunohistochemical staining for NeuN shows that the nuclei of the tumor cells are NeuN-positive; **b**: immunohistochemical staining for synaptophysin shows that the tumor cells and the neuropil-like islands are synaptophysin-positive; **c**: immunohistochemical staining for GFAP shows GFAP-negative tumor cells and trapped GFAP-positive reactive astrocytes; **d**: immunohistochemical staining for Ki-67 shows tumor cells with low proliferation index (about 2 %). **a**-**d** are at moderate magnification

The mass was solid-cystic with a size of about 8.0 × 7.0 cm. Hair, teeth, and bone tissues were found inside the mass. The solid region of the mass was relatively fragile, and yellow-white in color. Histological examinations showed mature epidermis, cutaneous appendages, adipose, bone, and neuroectodermal tissues; microscopic foci of immature cartilage tissues were also found in one visual field (low magnification). In another region, solid sheets and monomorphic round tumor cells were found, with capillary-sized blood vessels among the cells; the boundaries with surrounding neuroectodermal tissues were clear. The density of the tumor cells varied: neuropil-like islands were found in the loose-textured areas with low cell density. While in the compacted areas, moderate density cells with uniform round nuclei and perinuclear halos with oligodendroglioma-like honeycomb appearance were found. Nuclear mitosis, angiogenesis, and necrosis were not found (Fig. 2). Immunohistochemical staining showed that the tumor cells were synaptophysin- and NeuN- positive but GFAP- and CK-negative. The neuropil-like islands were also synaptophysin-positive. Trapped GFAP-positive reactive astrocytes were seen. The Ki-67 proliferation index was about 2 % (Fig. 3). Based on these findings, the patient was diagnosed with neurocytoma arising from mature ovary teratoma, with microscopic foci of immature cartilage tissues. The follow-up is needed to evaluate the prognosis.

## Discussion

Central neurocytoma is a rare CNS tumor that was first reported by Hassoun in 1982 [4]. It is mainly found in young adults, and the most commonly affected sites include the supratentorial lateral ventricle and/or the third ventricle, especially the foramen of Monro, therefore the tumor was named “central neurocytoma”. Central neurocytoma is composed of uniform round cells, and histological features include oligodendroglioma-like honeycomb appearance, perivascular fibrillar zones, and neuropil-like islands. The appearance of neuropil-like islands is considered to be a feature of tumor cells with neuronal differentiation. The tumor cells show strong immunoreactivity for synaptophysin, variable staining for NeuN [1, 2], whereas GFAP stains are typically negative, which further confirmed that they were differentiating into neurons. The Ki-67 proliferation index of the tumor cells is generally <2 % and the clinical course is usually benign, thus the current WHO classification assigns the central neurocytoma to grade II. Subsequently, tumors mimicking central neurocytomas but occurring within the cerebrum [5] and spinal cord [6, 7] were reported. Thus, the *WHO Classification of Tumors of the Central Nervous System* (4^th^ Edition) defined all extraventricular tumors with histological features and immunophenotypes similar to central neurocytoma as extraventricular neurocytoma, and classified them as WHO grade II tumor.

The features of the tumor in the present report were as follows: the tumor formed in the ovary of a 24-year-old female; part of the tumor appeared to be a mature teratoma with microscopic foci of immature cartilage tissues; sheet-like round tumor cells with homogenous morphology were found in other regions with oligodendroglioma-like honeycomb appearance and abundant neuropil-like islands. Furthermore, immunohistochemical staining showed that the tumor cells were synaptophysin- and NeuN-positive and GFAP-negative. Considering the differentia diagnosis against clear cell ependymomas and oligodendrogliomas, neuropil-like islands and diffuse synaptophysin and NeuN positivity are in favor of neurocytoma. A further differential diagnosis is a teratoma of the ovary with immature neuroepithelial elements. However, NeuN is not found in immature neural progenitor cells [8]. Therefore, the pathological diagnosis was a neurocytoma arising from a mature teratoma with microscopic foci of immature cartilage tissues.

Neurocytomas outside the CNS are very rare. Only three such cases have been reported to date [9–11], among which two cases arose from mature teratomas (in the ovary and adrenal gland, respectively), and the other occurred in the pelvis of a male patient. In all cases, at least one histological feature of neurocytoma was found; and more importantly, the tumor cells were positive for neuronal markers including synaptophysin and NSE, which confirmed that the tumors were neurocytoma outside the CNS. These patients were followed up for 6 months, 1 year, and 3 years, respectively, and no sign of recurrence was found. However, the prognosis remains unclear. Microscopic foci of immature cartilage tissues were found in one visual field (low magnification) in the present case, which was not reported in the previous cases. Thus, we recommended regular follow-up to our patient.

## Conclusion

In summary, neurocytoma outside the CNS rarely occurs. When pathological features similar to neurocytoma are found during the diagnosis of teratomas, the pathologists should carefully perform immunohistochemical examinations for confirmation and differentiation.

## Consent

Written informed consent was obtained from the patient for publication of this case report and accompanying images. A copy of the written consent is available for review by the Editor-in Chief of this Journal.
